# Effect of rosiglitazone on inflammatory cytokines and oxidative stress after intensive insulin therapy in patients with newly diagnosed type 2 diabetes

**DOI:** 10.1186/s13098-019-0432-z

**Published:** 2019-05-03

**Authors:** Juan Li, Xingping Shen

**Affiliations:** 1 Department of Emergency, Zhongshan Hospital Xiamen University, Xiamen, 361004 Fujian China; 2 Department of Endocrinology, Zhongshan Hospital Xiamen University, Xiamen, 361004 Fujian China

**Keywords:** Newly diagnosed type 2 diabetes mellitus (T2DM), Insulin sensitizer, Inflammatory cytokines, Oxidative stress

## Abstract

**Objective:**

To evaluate the effect of insulin sensitizer on inflammatory cytokines and oxidative stress in patients with newly diagnosed type 2 diabetes mellitus (T2DM).

**Methods:**

After intensive insulin therapy, patients with newly diagnosed T2DM were continuously treated with either insulin sensitizer or insulin for 48 weeks, and then their inflammatory cytokine and oxidative stress levels were measured.

**Results:**

Tumor necrosis factor alpha (TNF-α), interleukin (IL)-6, hypersensitive C reactive protein (hs-CRP), malondialdehyde (MDA), and 8-iso-prostaglandin F2α (8-iso-PGF_2α_) levels of the rosiglitazone (RSG) group and the rosiglitazone combined with metformin (RSG + metformin) group were significantly reduced after the treatments (*P* < 0.05). Hs-CRP, MDA, and 8-iso-PGF_2α_ levels of the metformin group were significantly reduced after the treatments (*P* < 0.05). Superoxide dismutase (SOD) and total antioxidant capacity (TAC) were significantly increased after the treatments in all three groups (*P* < 0.05 and *P* < 0.01).

**Conclusion:**

Early application of insulin sensitizers improved inflammation and oxidative stress in patients with newly diagnosed T2DM.

## Introduction

Patients with type 2 diabetes mellitus (T2DM) are at increased risk of cardiovascular diseases and associated clinical complications. It is also closely related to the severity of atherosclerosis [1, 2]. Traditional risk factors, such as dyslipidemia and hypertension, cannot fully explain the increase in the incidence of cardiovascular diseases in T2DM. Inflammation and oxidative stress play an important role in insulin resistance, T2DM, and cardiovascular complications. However, the relationship between inflammation, T2DM, and cardiovascular diseases is not well established [3]. T2DM is not only characterized by hyperglycemia but also involves low-grade inflammation. In addition, inflammation can be observed throughout the course of T2DM. Abnormal inflammation is a risk factor for cardiovascular disease in patients with T2DM.

A large number of inflammatory cytokines, such as tumor necrosis factor receptor 1 (TNF-R1), interleukin (IL)-1 beta (IL-1β), tumor necrosis factor alpha (TNF-α), and IL-6 are closely associated with the cardiovascular events in T2DM. Chronic hyperglycemia can induce oxidative stress, which inhibits insulin signaling pathways and induces endothelial dysfunction. Hence, to some extent, the pathogenesis of T2DM and the progress of vascular complications depend on the balance and regulation of oxidative stress in the body. Prevention and treatment of T2DM and its cardiovascular complications can be achieved by regulating the intrinsic and extrinsic factors of oxidative stress [4]. Oxidative stress and inflammation affect each other. Oxidative stress destroys adipose tissue to release adipocytokines such as TNF-α and IL-6α to trigger inflammation and is involved in the pathogenesis of insulin resistance [5]. Intensive insulin therapy reduces reactive oxygen species and reactive nitrogen formation in tissues and macrophages, thereby reducing inflammation [6]. A previous study showed that application of insulin sensitizer for 36 weeks in T2DM patients undergoing intensive insulin therapy alleviates vascular dysfunction and inflammation by reducing levels of free fatty acids and triglycerides and increasing adiponectin, and this effect is independent of glycemic control [7]. Rosiglitazone (RSG), an agonist of peroxisome proliferator-activated receptor gamma (PPARγ), regulates the transcription of multiple genes related to the effects of insulin and so significantly ameliorates insulin resistance and oxidative stress that helps lower the incidence of cardiovascular diseases in T2DM patients [8, 9]. In addition, RSG has remarkable pleiotropic activities in that it improves systemic inflammation status and endothelial function, and these allow it to exert cardiovascular benefits, which are independent of the improvement in blood glucose metabolism [10]. RSG has a significant anti-inflammatory effect. A previous study treated T2DM patients with RSG for 26 weeks and showed that RSG significantly reduces C-reactive protein expression, which is positively correlated with IL-6 expression, suggesting that it may affect inflammatory pathways through the transcriptional mechanism [11, 12].

Metformin treatment is well known to reduce the morbidity and mortality of cardiovascular diseases. However, to some extent, its cardio-protective effects are not associated with improvements in glycemic control or other risk factors, thereby increasing the likelihood that its pleiotropic effects will reduce the risk of cardiovascular diseases [13, 14]. Metformin can enhance insulin sensitivity by increasing the activity of insulin receptor tyrosine kinase, the number of insulin receptors, and affinity of insulin to target tissues, promoting the translocation of glucose transporter protein, inhibiting gluconeogenesis, and reducing hepatic glucose production. Metformin also reduces oxidative stress [13, 15]. Rosiglitazone and metformin are two types of insulin sensitizer with different mechanisms of action. They can be used alone or in combination for the treatment of T2DM. Their safety and clinical effect on insulin resistance have been extensively verified [16].

Comprehensive treatment with multiple drugs has been used for the treatment of T2DM. Although the overall survival of T2DM patients was not found to improve significantly, the number of cardiovascular events did show a significant decrease [17], but the exact mechanism remains unknown. A review article has shown that early intensive insulin therapy in patients with newly diagnosed T2DM significantly improves islet function and insulin sensitivity [18]. However, study of changes in inflammation and oxidative stress in patients with newly diagnosed T2DM who have received a 48-week treatment with insulin sensitizer after intensive insulin therapy has only rarely been reported. This study used RSG and RSG combined with metformin to treat patients with newly diagnosed T2DM for 48 weeks after the termination of intensive insulin therapy to compare their changes in inflammatory factors and oxidative stress to those of T2DM patients who continued to receive insulin therapy, thus evaluating the impact of early insulin sensitizers on the risk factors associated with vascular complications in patients with newly diagnosed T2DM.

## Research subjects and methods

### Research subjects

Six hundred eighty-six patients newly diagnosed with T2DM who had been admitted to the Department of Endocrinology in Zhongshan Hospital, Xiamen University, China from March 2012 to May 2017 and who had undergone a ≤ 1-year course of T2DM were enrolled in this study. They all received 14–21 days of intensive insulin pump therapy (insulin aspart) as the primary treatment, after which they were switched to oral antidiabetic drugs or continued subcutaneous injection of insulin aspart 30 for 48 weeks. They were randomly placed in the RSG (GlaxoSmithKline product, GlaxoSmithKline, Middlesex, UK) group, metformin group, RSG combined with metformin (RSG + metformin) group, and insulin aspart 30 group. All patients received medical nutrition guidance by phone. Diagnostic criteria of T2DM were in accordance with the World Health Organization (WHO) diagnostic criteria for diabetes proposed in 1999.

The inclusion criteria were newly diagnosed T2DM patients who had not received insulin or sulfonylurea treatments. The exclusion criteria were as follows: (1) patient age ≥ 70 years old; (2) positive for glutamic acid decarboxylase antibody (GADab) or islet cell antibody (ICA); (3) elevated creatinine levels (1.5 mg/dL for men and 1.4 mg/dL for women) and > 2-fold upper limit of liver enzymes; (4) serious coronary heart disease (myocardial infarction or acute angina) in the past 6 month and grade III–IV heart failure; (5) pregnancy or any smoking habits; (6) untreated proliferative diabetic retinopathy; (7) other autoimmune disease; (8) recent intake of angiotensin receptor blocker (angiotensin receptor blockers/ARB or angiotensin-converting enzyme inhibitor/ACEI) dilators, antibiotics, thiazolidinediones, statin-lowering lipids, or non-steroidal anti-inflammatory drug treatments; and (9) departure from the study area.

All patients received primary treatment using intensive insulin aspart pump for 14–21 days, followed by measurement of their fasting blood glucose and 2 h postprandial blood glucose. If the patients had < 7 mmol/L fasting blood glucose and < 10 mmol/L 2 h postprandial blood glucose, they were switched to oral antidiabetic drugs or subcutaneous injection of insulin aspart 30 for 48 weeks. Their glucose, insulin, C-peptide, lipids, liver function, inflammatory cytokines, and oxidative stress were measured before and after the 48-week treatment. In this study, 530 cases did not meet the inclusion criteria during the course of treatment, and only 156 cases were included in the final statistical analysis, including 40 cases in the RSG group, 39 cases in the metformin group, 36 cases in the RSG + metformin group, and 41 cases in the insulin aspart group. The research protocol was approved by the Ethics Committee of Zhongshan Hospital affiliated with Xiamen University, Fujian Province, China. All patients signed the written informed consent forms before participating in the study.

## Blood sampling and detections

The T2DM patients underwent plasma glucose, glycosylate hemoglobin, insulin C-peptide, islet cell antibodies, glutamic acid decarboxylase antibody, lipid, liver function, inflammatory cytokines, and pre- and post-prandial oxidative stress detection after the 14–21 days of intensive insulin therapy and then after the 48 weeks of different treatments.

In this study, Levels of fasting plasma glucose (FPG) were determined using the glucose oxidase method; glycosylate hemoglobin (hemoglobin A1c, HbA_lc_) levels were determined using high performance liquid chromatography; insulin levels were measured by radioimmunoassay; islet cell antibodies were detected using chemiluminescence; glutamic acid decarboxylase antibody (GADab) were determined using Immunoblotting test (Shenzhen Blot Biotech Co., Ltd, China); C-peptide levels were measured by electrochemiluminescence immunoassay; triglycerides were measured using an enzymatic colorimetric test (Roche Diagnostics, Basel, Switzerland); cholesterol analyses were performed using cholesterol oxidase assays. Blood levels of low-density lipoprotein-cholesterol (LDL-C) were determined by enzymatic clearance assay, and high-density lipoprotein cholesterol (HDL-C) levels were determined by HDL-C catalase assay. Aspartate aminotransferase (AST) and alanine transaminase (ALT) levels were determined using enzyme kinetic assays. Alkaline phosphatase (AKP), gamma-glutamyl transferase (γ-GT), and hypersensitive C reactive protein (hs-CRP) were performed using colorimetry, enzyme kinetic assay, and immune turbidimetric assay, respectively. IL-1β, TNF-α, and IL-6 levels were measured using ELISA kits purchased from Boster Biological Technology Co., Ltd. (Wuhan, China). The 8-iso-prostaglandin F2α (8-iso-PGF_2α_) levels were measured using an 8-iso-PGF_2α_ ELISA kit purchased from Nanjing Jiancheng Bioengineering Institute (Jiangsu Province, China). Superoxide dismutase (SOD), malondialdehyde (MDA), and total antioxidant capacity (TAC) were measured using kits purchased from Nanjing Jiancheng Bioengineering Institute (Jiangsu Province, China). Insulin resistance index of homeostasis model assessment (HOMA-IR) and islet β cell secretion of the homeostasis model assessment (HOMA-β) were calculated using the equations as follows: HOMA-IR = [FPG (mmo/L) × FINS (mU/L)]/22.5 and HOMA-β = [20 × FINS (mU/L)]/[FPG (mmo/L) − 3.5], respectively.

## Measurements of body mass index (BMI) and waist to hip ratio (WHR)

Once the patients’ condition became stable, their weight and height were measured (here given in kg and cm, respectively; with the accuracy of 0.1 kg and 0.1 cm, respectively) while they were not wearing shoes or hair accessories and wearing only underwear. Waist circumference (WC) was measured at the mid-point between the lower edge of rib cage and anterior superior iliac spine, and hip circumference was measured at the horizontal level of the greater trochanter of the femur (with an accuracy of 0.1 cm). The BMI and WHR were calculated using the following equations: BMI = bodyweight (kg)/height (m) [2] and WHR = WC/hip circumference.

## Statistical analysis

All data were processed and analyzed using SPSS 13.0 statistical software (SPSS Inc., Chicago, IL, US). Data from each group are presented as mean ± standard deviation (x ± s). Non-normally distributed data (e.g., HOMA-IR and HOMA-β) were log converted to normal distribution for analysis. Paired t-test was used to compare the difference before and after the treatments. ANOVA was used to compare multiple groups. Q-test was used to compare between every two groups. P < 0.05 was considered a statistically significant difference.

## Results

### Basic clinical conditions

In this study, bodyweight, BMI, fasting blood glucose, and 2-h postprandial blood glucose in the RSG group were significantly higher after the 48-week treatment (*P* < 0.05, *P* < 0.01); whereas HbA_1c_, HOMA-IR, and γ-GT were significantly lower (*P* < 0.05, *P* < 0.01). In the metformin group, fasting and 2-h postprandial blood glucose of the T2DM patients were significantly higher after the 48-week treatment (*P* < 0.05, *P* < 0.01); whereas bodyweight, BMI, HbA_1c_, HOMA-IR, triglyceride, and γ-GT were significantly lower after the 48-week treatment (*P* < 0.05, *P* < 0.01). In the RSG + metformin group, fasting and 2-h postprandial blood glucose of the T2DM patients were significantly higher after the 48-week treatment (*P* < 0.05, *P* < 0.01); whereas HbA_1c_, HOMA-IR, triglyceride, and γ-GT were significantly lower (*P* < 0.05, *P* < 0.01). In addition, after the 48-week treatment, HbA_1c_, fasting and 2-h postprandial blood glucose, and HOMA-IR in the RSG + metformin group were significantly lower than in the RSG group (*P* < 0.05). The 2-h postprandial blood glucose of the RSG + metformin group was significantly lower than that of the metformin group after the 48-week treatment (*P* < 0.05). In the insulin aspart 30 group, bodyweight and BMI after the 48-week treatment were significantly higher than that before the treatment and also higher than in the metformin group after the 48-week treatment (*P* < 0.05, *P* < 0.01); HbA_1c_ after the 48-week treatment was significantly lower than that before the treatment and also lower than in the RSG and metformin groups after the 48-week treatment (*P* < 0.05, *P* < 0.01); fasting and 2-h postprandial blood glucose of the insulin aspart 30 group were significantly lower than in the RSG and metformin groups (*P* < 0.05); and HOMA-IR was significantly higher than in the RSG and metformin groups (*P* < 0.05). No significant change in HOMA-β was observed in any of the four groups of T2DM patients following the 48-week treatment (*P* > 0.05, Table 1).

**Table 1:** Basic clinical conditions and biochemical parameters of the T2DM patients after intensive insulin therapy

	Rosiglitazone		Metformin		Rosiglitazone plus metformin		Insulin aspart 30	
	Baseline	48 weeks	Baseline	48 weeks	Baseline	48 weeks	Baseline	48 weeks
n	40	40	39	39	36	36	41	41
Age (years)	40.36 ± 10.02	40.36 ± 10.02	42.13 ± 9.54	42.13 ± 9.54	42.36 ± 9.52	42.36 ± 9.52	41.62 ± 9.87	41.62 ± 9.87
Sex (male/female)	21/19	21/19	23/16	23/16	20/16	20/16	24/17	24/17
Weight (kg)	69.57 ± 5.37	73.23 ± 9.55*	70.08 ± 11.15	66.34 ± 11.76*	72.69 ± 10.61	73.08 ± 9.38	69.22 ± 12.21	74.52 ± 11.63^△b^
BMI (kg/m^2^)	27.17 ± 2.74	29.55 ± 7.29*	28,32. ± 3.63	26.01 ± 6.71*	28.11 ± 6.85	28.28 ± 8.52	27.75 ± 7.62	30.63 ± 8.92*b
BP (mmHg)	132/76	134/80	136/80	138/76	132/78	130/76	134/78	138/80
HbA_1c_ (%)	13.41 ± 4.22	8.85 ± 2.66^△^	12.72 ± 5.85	7.97 ± 3.89^△^	15.82 ± 5.53	7.56 ± 3.95^△a^	13.58 ± 4.64	6.35 ± 3.06^△ab^
GAD	Negative	Negative	Negative	Negative	Negative	Negative	Negative	Negative
ICA	Negative	Negative	Negative	Negative	Negative	Negative	Negative	Negative
RSG (mg)	4	4	NA	NA	4	4	NA	NA
Metformin (g)	NA	NA	1.81 ± 0.89	1.89 ± 0.93	1.98 ± 0.56	2.05 ± 0.83	NA	NA
Glucose_Fasting_ (mmol/L)	6.72 ± 2.39	8.48 ± 2.42*	6.44 ± 3.16	7.39 ± 3.54 *	6.63 ± 3.84	7.54 ± 2.13*^a^	6.68 ± 2.58	6.85 ± 3.66^ab^
Glucose_2-hour postprandial_ (mmol/L)	8.58 ± 4.46	13.50 ± 3.76^△^	8.05 ± 4.17	13.72 ± 4.48^△^	8.96 ± 2.63	10.03 ± 3.89^△ab^	8.93 ± 3.74	9.48 ± 3.73^ab^
C-peptide_Fasting_ (ng/mL)	3.73 ± 1.01	4.66 ± 2.63	4.03 ± 2.22	3.96 ± 1.57	3.88 ± 1.95	3.99 ± 1.05	4.14 ± 1.36	4.01 ± 1.74
Insulin_Fasting_ (mIU/L)	18.62 ± 10.52	16.76 ± 8.59	19.70 ± 11.15	17.34 ± 9.56	17.25 ± 7.23	16.64 ± 6.53		
HOMA-IR	6.92 ± 2.14	5.89 ± 3.25*	7.11 ± 3.35	5.04 ± 2.31*	6.59 ± 3.28	4.72 ± 3.68^△a^	7.56 ± 3.45	6.93 ± 3.72^ab^
HOMA-β	48.26 ± 20.15	47.27 ± 15.13	49.37 ± 18.81	48.48 ± 19.64	50.38 ± 30.58	49.49 ± 26.10	48.64 ± 18.63	49.53 ± 16.68
TG (mmol/L)	1.53 ± 0.96	1.56 ± 0.87	1.67 ± 0.78	1.36 ± 0.99*	1.92 ± 1.08	1.24 ± 0.85*	1.89 ± 0.75	1.68 ± 0.84
CH (mmol/L	5.63 ± 2.63	5.08 ± 3.95	5.92 ± 3.33	5.35 ± 3.45	5.41 ± 2.52	5.20 ± 2.47	5.57 ± 3.84	5.67 ± 2.68
LDL (mmol/L)	2.48 ± 1.58	2.17 ± 2.56	2.96 ± 1.99	2.63 ± 2.02	2.78 ± 2.22	2.05 ± 2.65	2.53 ± 1.56	3.32 ± 1.46
HDL (mmol/L)	0.85 ± 0.37	0.88 ± 0.62	0.95 ± 0.64	0.98 ± 0.35	0.97 ± 0.84	0.90 ± 0.76	0.87 ± 0.45	0.98 ± 0.56
ALT (U/L	48.20 ± 9.35	46.09 ± 7.64	46.64 ± 10.24	48.67 ± 8.28	47.11 ± 5.69	45.87 ± 8.47	47.65 ± 10.75	46.56 ± 8.47
AST (U/L)	42.07 ± 10.84	39.53 ± 8.78	44.33 ± 11.58	42.16 ± 10.93	41.33 ± 8.55	39.52 ± 9.19	43.64 ± 7.76	42.58 ± 10.76
AKP (U/L)	98.66 ± 6.87	97.23 ± 8.30	96.47 ± 7.77	98.54 ± 9.39	97.77 ± 7.22	95.65 ± 7.46	97.64 ± 8.74	98.75 ± 10.75
γ-GT (U/L)	58.65 ± 5.11	42.94 ± 8.28 *	59.26 ± 7.87	49.51 ± 9.67 *	57.09 ± 6.43	40.10 ± 9.33*	59.46 ± 6.59	58.45 ± 7.55

* *P* < 0.05, compared with the baseline level

^△^*P* < 0.01, compared with the baseline level

^a^*P* < 0.05, compared with the rosiglitazone group after treatment

^b^*P* < 0.05 compared with the metformin group after treatment

### Changes in inflammatory cytokines and oxidative stress before and after treatment in T2DM patients

Comparison of inflammatory cytokines and oxidative stress in the T2DM patients before and after the 48-week treatments showed that TNFα, IL-6, hs-CRP, MDA, and 8-iso-PGF_2α_ of the RSG group were significantly reduced after the 48-week treatment (*P* < 0.05); whereas SOD and TAC of the RSG group significantly increased after the 48-week treatment (*P *< 0.05); no significant changes in IL-1β were found in the RSG group after the 48-week treatment (*P* > 0.05). In the metformin group, SOD and TAC were significantly higher after the 48-week treatment (*P* < 0.05, *P* < 0.01); whereas hs-CRP, MDA, and 8-iso-PGF_2α_ were significantly lower after the treatment (*P* < 0.05). There was no significant change in IL-6 and IL-1β in the 48-week metformin treatment group (*P* > 0.05). In the RSG + metformin group, TNFα, IL-6, hs-CRP, MDA, and 8-iso-PGF_2α_ were significantly reduced after the 48-week treatment (*P *< 0.05); while SOD and TAC significantly increased (*P* < 0.05, *P *< 0.01). IL-6, hs-CRP, and 8-iso-PGF_2α_ after the 48-week RSG + metformin combination therapy were significantly lower than after 48-week of treatment with RSG alone (*P* < 0.05); in contrast, SOD in the RSG + metformin group was significantly higher than in the RSG group after the 48-week treatment (*P* < 0.05). No significant difference in IL-1β was observed after the 48-week RSG + metformin treatment (*P* > 0.05). MDA and 8-iso-PGF2α in the insulin aspart group were significantly reduced, while SOD and TAC significantly increased (*P* < 0.01, *P* < 0.05) after the 48-week treatment. There was no significant change in TNFα, IL-6, hs-CRP, and IL-1β after the 48-week insulin aspart treatment (*P* > 0.05) (Table 2).

**Table 2:** Changes in inflammatory cytokines and oxidative stress after the intensive insulin therapy in T2DM patients (x ± s)

	Rosiglitazone		Metformin		Rosiglitazone plus metformin		Insulin aspart 30	
	Baseline	48 weeks	Baseline	48 weeks	Baseline	48 weeks	Baseline	48 weeks
TNFα (pg/mL)	28.57 ± 10.37	22.12 ± 11.66*	30.24 ± 15.78	28.33 ± 12.34	27.91 ± 12.21	21.59 ± 9.82*	28.75 ± 13.84	27.87 ± 10.22
IL-6 (pg/mL)	8.13 ± 2.22	6.68 ± 3.69*	9.21 ± 3.12	8.79.45 ± 4.01	8.88 ± 3.91	5.13 ± 3.93*^a^	8.29 ± 4.63	7.31 ± 4.35
Il-1β (ng/mL)	3.65 ± 1.63	3.71 ± 1.98	3.93 ± 2.95	3.52 ± 2.07	3.78 ± 2.26	4.02 ± 1.99	3.65 ± 2.62	4.83 ± 1.59
hs-CRP (mg/L)	4.45 ± 1.51	3.24 ± 1.96*	4.90 ± 1.98	3.01 ± 1.77*	4.86 ± 1.70	2.23 ± 1.57*^a^	4.67 ± 1.37	4.11 ± 1.18
SOD (kU/L)	72.62 ± 18.56	76.36 ± 20.26*	76.32 ± 20.21	80.55 ± 25.27^△^	74.98 ± 19.50	79.24 ± 23.70^△a^	75.46 ± 21.56	78.68 ± 22.33^△^
MDA (μmol/L)	6.03 ± 1.42	4.28 ± 1.94*	6.94 ± 1.68	5.22 ± 1.73*	6.53 ± 1.25	4.98 ± 1.56*	6.14 ± 1.88	4.38 ± 1.62*
8-iso-PGF_2α_ (μg/L)	10.35 ± 4.44	8.87 ± 5.91*	12.11 ± 7.66	7.65 ± 7.15*	11.30 ± 6.80	7.22 ± 6.31*^a^	10.87 ± 6.83	8.94 ± 6.34*
TAC (kU/L)	24.62 ± 10.69	27.30 ± 12.35*	25.31 ± 12.24	28.97 ± 9.18*	23.66 ± 11.01	27.00 ± 10.82*	24.48 ± 12.47	28.83 ± 10.82*

* *P* < 0.05, compared with the baseline level

^∆^*P* < 0.01, compared with the baseline level

^a^*P* < 0.05, compared with the RSG group after treatment

## Discussion

This study demonstrated that metformin monotherapy after intensive insulin therapy significantly reduced the overall bodyweight and BMI and improved insulin resistance and dyslipidemia in T2DM patients. This was consistent with the findings reported in a previous study [19]. Studies have shown that thiazolidinediones (TZDs) can reduce the body weight of T2DM patients by 2–3% for each percentage of HbA_1c_ [20, 21]. This study also showed that RSG monotherapy significantly increased the body weight and BMI of T2DM patients with increased insulin sensitivity and improved glycemic control. These may have been due to RSG stimulation on the appestat, which improved appetite and led to elevated body fluid and plasma volume in T2DM patients, increasing body weight. Nevertheless, there was no increase in heart failure, peripheral edema, or dyspnea in the RSG-treated T2DM patients in this study, suggesting that RSG changed the body weight of T2DM patients without causing macrovascular or cardiac dysfunction. Moreover, RSG-induced weight gain might also be associated with increased subcutaneous fat and reduced visceral fat in the patients. Changes in fat distribution due to decreases in visceral fat area and the ratio of visceral-to-subcutaneous fat may also explain the overall weight gain and improvement of blood glucose control in the T2DM patients [22].

In this study, no change in body weight or BMI was observed in the T2DM patients after the RSG + metformin combination therapy, which may have been due to the reduction in body weight associated with metformin. This combination therapy reduced visceral fat by increasing insulin sensitivity, decreasing free fatty acid production, and increasing subcutaneous fat [23] to lower triglycerides and fatty acid production. In addition, the RSG + metformin combination therapy significantly reduced HbA_1c_, fasting and 2 h postprandial blood glucose, and HOMA-IR, which were significantly lower than after the RSG monotherapy, suggesting that the combination therapy had a synergistic effect on increasing insulin sensitivity and reducing blood sugar. Although HbA_1c_ was reduced in the T2DM patients with oral drug administration of RSG, metformin, or RSG + metformin following intensive insulin therapy, their fasting blood glucose and postprandial blood glucose remained elevated, suggesting that treatment with only metformin and insulin sensitizer did not efficiently control the blood glucose and that addition of other drugs or insulin are necessary. Continued application of insulin aspart after intensive insulin therapy was associated with better glycemic control in T2DM patients even though insulin sensitivity did not change much after the treatment as compared with before the treatment, which may have been due to the absence of insulin sensitizer, and the body weight of the patients increased after insulin therapy.

TZD has attracted attention in the intervention of T2DM, metabolic syndrome, cardiovascular diseases, and inflammation. A recently study has shown that TNF-α and IL-6 are independently associated with CHD risk, in an approximately log-linear manner [24]. TNF-α plays a key role in damaging macrovascular and microvascular circulation [24]. Due to possible interference from TNF-α with respect to insulin signaling, TNF-α is considered a causative agent in the pathogenesis of obesity-associated insulin resistance and T2DM [25]. In this study, TNF-α levels were significantly reduced following RSG or RSG + metformin treatment, while no change in TNF-α was observed after the metformin treatment, suggesting that RSG might be associated with the activation of peroxisome proliferator-activated receptor gamma (PPAR-γ), reduction of the efficiency of fatty acid production in liver, and reduction of TNF-α release by adipocytes [26]. One in vitro study showed that metformin concentrations up to 10 μM can inhibit lipopolysaccharide (LPS)- or oxidized LDL (oxLDL)-stimulated TNF production by human monocytes [27]. However, use of 1000 and 2000 mg metformin in healthy individuals resulted in 8.7 μM and 13 μM plasma concentration, respectively [28]. In the present study, although TNF-α level in T2DM patients was lower after metformin monotherapy, no statistically significant results were observed. It has not been established whether this is associated with insufficient dosage of metformin (< 2000 mg in the study), and extended treatment will be necessary to address this question. Continued insulin aspart treatment after the intensive insulin therapy did not change TNF-α levels in T2DM patients, which might be related to obesity, lifestyle, and uncontrollable genetic factors.

Interestingly, IL-6 acts as a proinflammatory and anti-inflammatory factor simultaneously, depending on the target tissue and metabolic status. IL-6, a proinflammatory cytokine, is a co-inducible factor that can lead to obesity-related IR, which is a prerequisite for T2DM development. However, the identification of IL-6 as a myokine, a protein produced and secreted by skeletal muscle to exert paracrine or endocrine roles in the insulin-sensitizing effects following exercise [29]. For this reason, IL-6 is considered a pleiotropic cytokine involved in normal function of the immune system, hematopoiesis, metabolism, and the pathogeneses of metabolic disorders and cardiovascular diseases. The impact of increased IL-6 in T2DM patients on glucose metabolism and insulin sensitivity remains controversial [30]. Plasma IL-6 levels can be used to predict macrovascular events and death in T2DM patients if used alongside cardiovascular events and other risk factors [31]. In this study, we showed that IL-6 levels of the T2DM patients in the RSG and RSG + metformin groups were reduced following the treatments, which was consistent with findings reported by Kadoglou et al. and Erem et al. [32, 33]. The significant reduction in IL-6 levels after the RSG + meformin combination therapy might be associated with metformin-induced increases in insulin sensitivity and the activation of AMP-activated protein kinase (AMPK), which prevented nuclear factor-Kappa beta (NF-κB) from reducing the release of IL-6 [13]. However, discontinuation of insulin after intensive insulin therapy while switching to metformin monotherapy did not change the IL-6 levels in the T2DM patients in this study. This result was inconsistent with previous studies [34], which might be due to the pleiotropic effects of IL-6 or the dosage or treatment duration of the metformin therapy [35]. This should be confirmed by further study.

IL-1β promotes insulin resistance in tissues, thereby affecting distal vascular wall, skeletal muscle, myocardium, and kidney. It is closely associated with atherosclerotic processes and obesity-related inflammation [36]. In addition, IL-1β participates in the autoimmunity of T2DM islet cells by activating T cells, B cells, and macrophages and increasing β cell antigen expression and apoptosis. In this study, no changes in IL-1β levels were observed. This result was consistent with the data reported by Mooradian et al. [37].

CRP is an acute-phase reactant and a systemic sensitive marker of inflammation and tissue damage. One recent study has shown that hs-CRP is an independent predictor of heart failure in T2DM [38]. Another previous study showed that RSG treatment results in a rapid and sustained reduction in CRP, which is not associated with insulin sensitivity, HbA_1c_, or weight gain. Metformin treatment moderately and gradually reduces CRP, which may be partially related to the changes in body weight but is independent of glycemic control or insulin sensitivity [39]. In this study, hs-CRP levels were lower in T2DM patients after RSG, metformin, and RSG + metformin treatments. This might be associated with the improved adipose tissue metabolism and regulation of adipocyte endocrine function by prolonged application of RSG, as well as the activation of nitric oxide synthases by metformin to inhibit CRP production in the liver. These results were consistent with the findings reported in previous studies [13, 39]. Treatment with RSG resulted in a rapid reduction in CRP that occurred as early as 2 weeks after initiation of treatment, well before the full effects of TZDs on glucose-lowering, lipid profile changes, and weight gain were manifested. This temporal difference between changes in CRP and those in metabolic markers suggests that an improvement in adipose tissue metabolism could be a prelude for other, later metabolic changes. Unlike with RSG treatment, the reduction in CRP in the metformin groups was small and gradual over time [39]. Recent studies have shown that metformin reduces the adverse effects of CRP by reducing the expression of genes related to innate immune responses and inflammation [13]. Although previous studies have reported that short-term insulin therapy can reduce hs-CRP levels [40], no changes in hs-CRP were found after the intensive insulin therapy and the subsequent 48-week insulin aspart treatment. This is consistent with the findings reported in a previous study [41]. We suspect this might be related to the increase in BMI and the application of exogenous insulin, which increased the hs-CRP level [42].

Free radical aggregation in the blood vessels of T2DM patients can activate harmful biochemical pathways, leading to vascular inflammation and the production of reactive oxygen species (ROS). Our previous study has shown that oxidative stress is already taking place in pre-diabetes (Pre-T2DM) [43] and plays an important role in microvascular and macrovascular complications in T2DM. Although the effect of RSG on oxidative stress has been controversial, most scholars believe that this controversy is related to RSG dosage, concentration, duration of treatment, and experimental method [44]. One previous study has shown that RSG reduces NADPH-stimulated production of superoxide anion in rat arteries, thereby reducing oxidative stress [45]. PPAR-γ activation in in vitro vascular endothelial cells reduces superoxide anion production and NADPH oxidase expression and promotes nitric oxide production through PPAR-γ-dependent mechanism [46]. However, the specific mechanism by which TZD reduces oxidative stress remains unclear. Metformin has a pleiotropic effect on cardiovascular protection independent of hypoglycemic activity and is involved in the reduction of ROS production as a mild inhibitor of mitochondrial respiratory complex I [13]. In this study, metformin and RSG treatments after intensive insulin therapy significantly improved the SOD and 8-iso-PGF2α levels in the T2DM patients. These improvements were more pronounced in the T2DM patients receiving RSG + metformin combination therapy. MDA and TAC were improved in T2DM patients after metformin and RSG monotherapies. We here speculate that metformin and RSG have a synergistic effect on reducing oxidative stress. This study showed that continued insulin aspart treatment in T2DM patients after intensive insulin therapy relieved oxidative stress to a significant extent, and its mechanism might involve insulin, which improved mitochondrial function and suppressed cytokines to further alleviate oxidative stress [47]. This result was consistent with the findings reported in a previous study [48]. In clinical practice, cardiovascular disease in patients with T2DM has not been eradicated by intensive glycemic control combined with multifactorial therapy. In this way, therapeutic strategies based on the pathogenesis will be necessary, and insulin sensitizer should be one of the options available for consideration [49]. In addition to regulating blood sugar, insulin also plays an important role in immunomodulation and inhibition of oxidative stress. Intensive insulin therapy alone ameliorates oxidative stress [48]. Continued use of insulin or regimens including TZD and metformin after intensive insulin therapy have been found to promote sustained improvement in oxidative stress in patients with newly diagnosed T2DM. Further studies will be necessary to evaluate its effects on macrovascular complications in T2DM.

In summary, the thiazolidinedione rosiglitazone was previously shown to increase the risk of myocardial infarction and cardiovascular mortality [50], but following the extensive monitoring by the FDA, no new adverse safety data has been demonstrated [51], early and prolonged application of TZD and metformin after intensive insulin therapy in patients with newly diagnosed T2DM had a specific effect on improving inflammatory status and oxidative stress in these patients. However, further studies will be necessary to confirm whether such therapeutic strategies help to prevent or reduce cardiovascular complications because TZD increases the risk of heart failure. Given that this study is limited to the treatment groups and had a small sample size and no control group, further studies with larger sample sizes, involvement of more patients with cardiovascular risk factors and cardiovascular disease, and proper control groups will be necessary to validate our findings.

## Abbreviations

T2DM: type 2 diabetes mellitus
TNF-α: tumor necrosis factor alpha
IL-6: interleukin
hs-CRP: hypersensitive C reactive protein
MDA: malondialdehyde
8-iso-PGF2α: 8-iso-prostaglandin F2α
RSG: rosiglitazone
SOD: superoxide dismutase
TAC: total antioxidant capacity
TNF-R1: tumor necrosis factor receptor 1
IL-1β: interleukin-1beta
TNF-α: tumor necrosis factor alpha
IL-6: interleukin-6
PPARγ: peroxisome proliferator-activated receptor gamma
GADab: glutamic acid decarboxylase antibody
ICA: islet cell antibody
HbA_lc_: hemoglobin A1c
LDL-C: low-density lipoprotein-cholesterol
HDL-C: high-density lipoprotein cholesterol
AST: aspartate aminotransferase
ALT: alanine transaminase
AKP: alkaline phosphatase
γ-GT: gamma-glutamyl transferase
HOMA-IR: insulin resistance index of homeostasis model assessment
HOMA-β: islet β cell secretion of the homeostasis model assessment
BMI: body mass index
WHR: waist to hip ratio
LPS: lipopolysaccharide
oxLDL: oxidized LDL
